# Experimental and Numerical Investigation of a Lattice Structure for Energy Absorption: Application to the Design of an Automotive Crash Absorber

**DOI:** 10.3390/polym14061116

**Published:** 2022-03-10

**Authors:** Carlo Boursier Niutta, Raffaele Ciardiello, Andrea Tridello

**Affiliations:** Department of Mechanical and Aerospace Engineering, Politecnico di Torino, 10129 Turin, Italy; raffaele.ciardiello@polito.it (R.C.); andrea.tridello@polito.it (A.T.)

**Keywords:** lattice structure design, energy absorption, additive manufacturing, fused deposition modeling

## Abstract

In this work, an experimental and numerical analysis of a lattice structure for energy absorption was carried out. The goal was to identify the most influencing parameters of the unit cell on the crushing performances of the structure, thus guiding the design of energy absorbers. Two full factorial plans of compression tests on cubic specimens of carbon nylon produced by fused deposition modeling (FDM) were performed. The factors were the beam diameter and the number of unit cells. In the first factorial plan, the specimen volume is constant and the dimensions of the unit cell are varied, while the second factorial plan assumes a constant size of the unit cell and the volume changes in accordance with their number. The results showed that the specific energy absorption increases with the diameter of the beam and decreases with the size of the unit cell. Based on these results, a crash absorber for the segment C vehicle was designed and compared with the standard component of the vehicle made of steel. In addition to a mass reduction of 25%, the improved crushing performances of the lattice structure are shown by the very smooth force-displacement curve with limited peaks and valleys.

## 1. Introduction

Lattice structures are three-dimensional open-celled structures that are topologically ordered and composed of repeating unit cells [1,2,3]. The use of these structures for industrial applications and research activities has strongly increased in the last 10 years because of the recent advancements in additive manufacturing (AM) technologies. Indeed, these structures cannot be easily fabricated with traditional manufacturing technologies [3]. Lattice structures present outstanding potentialities since an optimum balance of stiffness, strength, and static or dynamic behavior can be achieved by topologically combining the cells to obtain an engineered response to a specific structural problem [4]. The mechanical behavior of lattice structures depends on the density, unit-cell size, geometrical configurations, aspect ratio, and the rate of loading [5]. Thus, different material properties can be obtained by designing the spatial configuration of the cells and their diameters. These structures can be easily produced with fused deposition modeling (FDM). Many studies [6,7,8,9,10,11] have compared the mechanical behaviors of lattice structures to cellular materials (or foams) since the only difference is that lattice structures present a regular repeating structure of their unit cells. Unlike cellular structures that present random-oriented unit cells, the topological position of lattice structures can be preliminarily designed to model the mechanical components and optimize the mechanical performance. This characteristic makes the lattice structures appropriate for energy absorption applications since the collapsing behavior is controlled [12,13,14]. Hasan et al. [12] studied the mechanical behavior under impact of the sandwich titanium structures with honeycomb and lattice cores. The mechanical performances of the two cores were quite similar in terms of absorbed energies. However, the lattice core presented a damaged zone that was more localized compared to that of the sandwich structures. McKnown et al. [13] reported a static and dynamic experimental analyses of steel lattice structures with two different architectures (body-centered cubic, BCC, with and without vertical support). They showed that the addition of the vertical strut to the BCC architecture could at least double the values of the absorbed energy under quasi-static and dynamic conditions. Although lattice structures made with metals have been widely investigated [6,7,8,9,10,11,12,13], polymer-based lattice structures are gaining high attention due to the high specific strength and impact properties [14]. Furthermore, reinforced polymers also enable the possibility of lightweight and smoothen the deceleration curve under impact crash [15]. Habib et al. [16] studied the energy absorption mechanism of different lattice architectures made of polyamide under quasi-static loads. The study [16] showed that, among the six different structures, an octagonal lattice structure presents the largest energy absorption characteristic. Furthermore, the properties of the polymer lattice structures were compared with traditional syntactic foams. They found that the mechanical behavior under compression of the octagonal lattice structures was superior in terms of absorbed energy and crushing response compared to stochastic foams. Davami et al. [17] studied the crushing behavior of lattice structures made with two different thermoplastic polymers: ABS and PMMA. They observed that the ABS-based polymer was able to absorb higher energy (30%) during the crush and the two materials exhibited a high recovery shape. The authors [17] showed that these two materials presented mechanical properties that were similar to the materials that are traditionally used for components used as energy absorbers.

More recently, Santiago et al. [18] used lattice structures made in PEEK materials for high temperature applications such as custom implants or aerospace structures. Although the study points to high temperature applications, it reports a preliminary activity on a small lattice structure made of amorphous PEEK, semi-crystalline PEEK, carbon fiber PEEK, and PEEK lattices based on an octet configuration. The study showed that the semi-crystalline PEEK resulted in the highest compressive strength of 21 MPa and modulus. Weeks et al. [19] studied the strain-rate compression behavior of polymeric rod and plate lattice structures. The study showed that the failure strength, stiffness, and specific energy absorption of lattice specimens were dependent on the strain-rate and on the relative density of the cell. The experiments carried out at different strain-rates on lattice specimens and on the base material also suggest that the observed dynamic effects of hardening, stiffening, and decreased fracture strain can be attributed to the polymeric material. Della Ripa et al. [20] carried out an experimental and numerical analysis on the crushing behavior of lattice structures made of carbon nylon filament. The authors experimentally assessed different lattice structures reported in the literature and determined a proper architecture that optimizes the crushing behavior. Furthermore, they found that the use of the 1-D elements in finite element (FE) models can greatly reduce the simulation time without significantly affecting the accuracy.

In conclusion, many works [14,15,16,17,18,19,20,21,22,23] have shown that lattice structure can be opportunely designed and manufactured through the AM technique to obtain unprecedented properties in the energy absorption. The reader can refer to Tamburrino et al. [24] for a recent extensive literature review on the design process of additively manufactured lattice structures. The review [24] provides a guide for researchers and engineers in the design of lattice structures, discussing approaches and strategies currently available. In particular, unit cell types, sizes, densities, and structures were taken into consideration. Even though many works in the literature [14,15,16,17,18,19,20,21,22,23] have addressed the mechanical behavior of lattice structures made with thermoplastic polymers, reinforced and not, a comprehensive study that experimentally analyzes the mechanical behavior of lattice structures and applies these results for the design of a component subjected to crash has not been reported and was the aim of this work.

In the current work, the lattice cell, preliminary studied in [20], was analyzed because of its high energy absorption properties. In particular, the influence of the beam and of the number of cells were investigated. Compression tests were carried out to determine the mechanical properties and the energy absorption capabilities of these structures. An analysis of variance (ANOVA) was used to stochastically investigate the influence of the diameters of the lattice structures, the number of repeated cells, and their interactions. FE models of the compression tests performed on the lattice structures were created and validated through a comparison with the experimental load–displacement curves. Finally, the validated model of lattice structure that presented the highest mechanical performances was used to analyze the mechanical behavior of a cash box made with the same materials. The results obtained with this simulation were compared with the mechanical behavior of a metal cash box already analyzed in the literature [15]. The current work shows that the crash box made with a lattice structure presented a smoother deceleration that is beneficial for the occupants of the vehicle during the crash and a weight reduction of 25% compared to the steel component.

## 2. Materials and Methods

In this section, the investigated carbon nylon material used for manufacturing the specimens and the experimental activity are described. In Section 2.1, the properties of the carbon nylon filament and the process parameters selected for printing the specimens are reported. In Section 2.2, the geometry of the lattice structure specimens used for the experimental tests and the testing plan are analyzed, while, in Section 2.3, the testing setup for the compression tests is described in detail. Finally, in Section 2.4, the numerical model of the lattice structure is presented.

### 2.1. Carbon Nylon Properties and Printing Parameters

In this section, the material used to manufacture the specimens is described. Originally, according to [20], the aluminum alloy AlSi10Mg was considered for the design of components with high absorbing capabilities made of lattice structures to be produced with a selective laser melting (SLM) process. However, the analysis of the experimental results in [20] suggested the use of carbon nylon, characterized by high specific energy absorption. Accordingly, carbon nylon was considered to manufacture the specimens and for the subsequent design of the crash box in this paper. It is worth noting that metal alloys such as the AlSi10Mg alloy can be used for the design of parts with high absorbing capabilities in lattice structures. In this case, the process parameters and the post processes such as heat-treatments should be optimized to increase the absorbing capabilities of the designed lattice structure part. On the other hand, a metal part in lattice structures for energy absorption applications can be characterized by a larger cost.

A Fabbrix^®^ carbon nylon filament with a 2.85 mm diameter was employed to produce the specimens with a FDM process and by using an Ultimaker 2+ Connect, equipped with a 0.4 mm Olsson Ruby nozzle. The process parameters suggested by the carbon nylon filament producer were considered to manufacture the specimens. The building plate and the nozzle temperature were set to 60 °C and to 250 °C, respectively, with a printing speed of 40 mm/s. Preliminary analyses were also carried out to optimize the whole set of process parameters such as the wall thickness or the infill pattern [20]. In particular, the process parameters were optimized in order to limit the difference between the nominal diameter of the lattice structure beams and the diameter experimentally measured by using an optical microscope to less than 10%. At most, two specimens were printed together on the building platform to limit the possible scatter of the mechanical properties due to the specimen position on the building platform.

The specimen geometries in the *stl* format were created with the software CREO^®^ and the Ultimaker Cura software was used for the slicing process. The mechanical properties of the printed carbon nylon used for the numerical simulations were retrieved from the filament producer [25,26], where the results of the experimental tests are available.

### 2.2. Lattice Structure Specimens Geometry and Testing Plan

**Figure 1:**
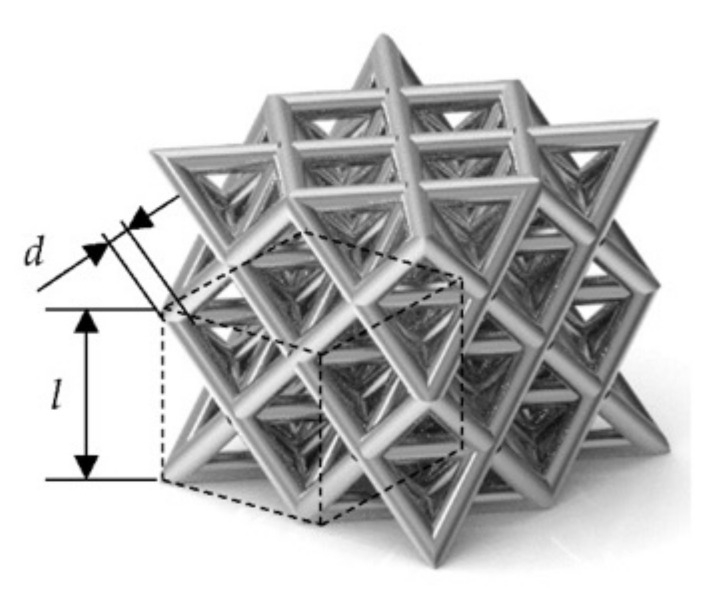
Example of the specimens subjected to compression tests with the cell unit cell geometry highlighted.

In [20], the cell geometry ensuring the highest absorbing capability was experimentally assessed by comparing different cell geometries available in the literature. In this paper, the cell geometry selected in [20] was considered, focusing on the influence of the cell parameters and the number of unit cells on the absorption capability. Figure 1 shows a rendering of a tested specimen, with the unit cell geometry highlighted. In the figure, *d* represents the beam diameter, whereas *l_cell_* represents the cell size.

The experimental activity was carried out according to a factorial design scheme, in order to investigate the influence of the cell parameters on the absorbing capabilities. In particular, the influence of the beam diameter d (Factor A) and the number of cells n (Factor B) was investigated. Table 1 summarizes the levels considered for Factor A and Factor B. With number of cells, the authors refer to the number of cell repetition in order to obtain a cubic specimen. For the sake of clarity, with *n* = 2, the authors refer to a cubic specimen with 2 × 2 × 2 unit cells (i.e., with two cells for each side of the base and two cells along the height). The levels of factor A were chosen by considering the manufacturing limits and constraints. Indeed, a beam diameter of 0.9 mm is the smallest that can be produced with acceptable quality and limited tolerances. In contrast, with d larger than 1.8 mm, the unit cell tends to a cell with a 100% infill and with limited voids (i.e., no more a lattice structure), thus of no interest in this analysis. The values reported in brackets refer to the actual diameters measured on the printed specimens through an optical microscope. The discrepancy with the nominal values can be attributed to the manufacturing process and to the precision of the printing machine.

The following multilevel factorial design was experimentally followed to investigate the influence of Factors A and B. In the following, the tests were identified with the notation *AxBy*, with *x* and *y* referring to the level of Factors A (4 levels) and B (3 levels), according to Table 1. For each combination of factors and levels, two specimens were manufactured and tested.

This experimental scheme was repeated twice. In the first experimental analysis, called factorial plan 1 (*FP1*), the total volume of the specimen was fixed and kept constant to 16.58 cm^3^ (i.e., the volume of a cube with 25.5 mm side). Accordingly, by varying the levels of Factor B, the cell size can be varied. This analysis, therefore, permits us to assess the influence of the cell size, together with the cell diameter, on the absorbing capabilities of parts made of lattice structures. In the following, for *FP1*, Factor B was assumed to correspond to the cell size, *l_cell_*, with levels 12.75 mm (B = 1), 8.35 mm (B = 2), and 6.38 mm (B = 3).

**Figure 2:**
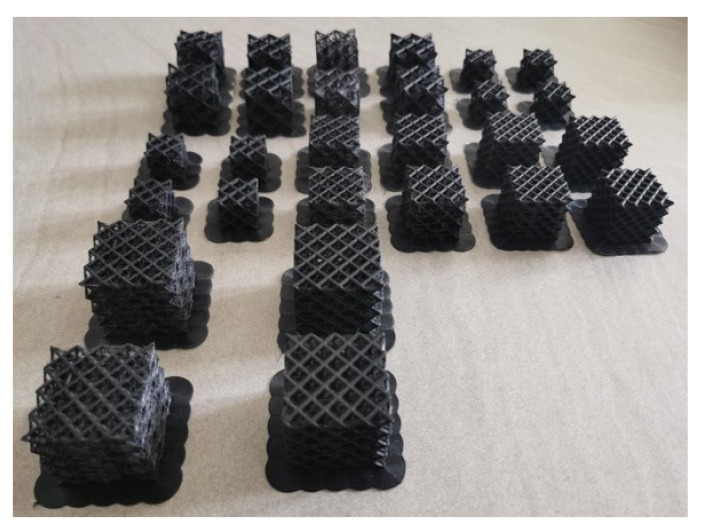
Twenty-eight out of 48 carbon nylon specimens for the compression tests.

In the second analysis, called factorial plan 2 (FP2), the cell size was kept constant and equal to that defined in [20] and the number of cells was increased with no volume constraints, enabling us to understand whether the absorbing capabilities vary with the number of cells of the specimen. This analysis, together with that carried out in FP1, is fundamental when components made of lattice structures are designed, since many cells are repeated and the design is generally carried out by considering the energy-absorbing properties of a single cell. Figure 2 shows 28 out of 48 specimens produced for the experimental tests.

### 2.3. Compression Tests: Experimental Setup

**Figure 3:**
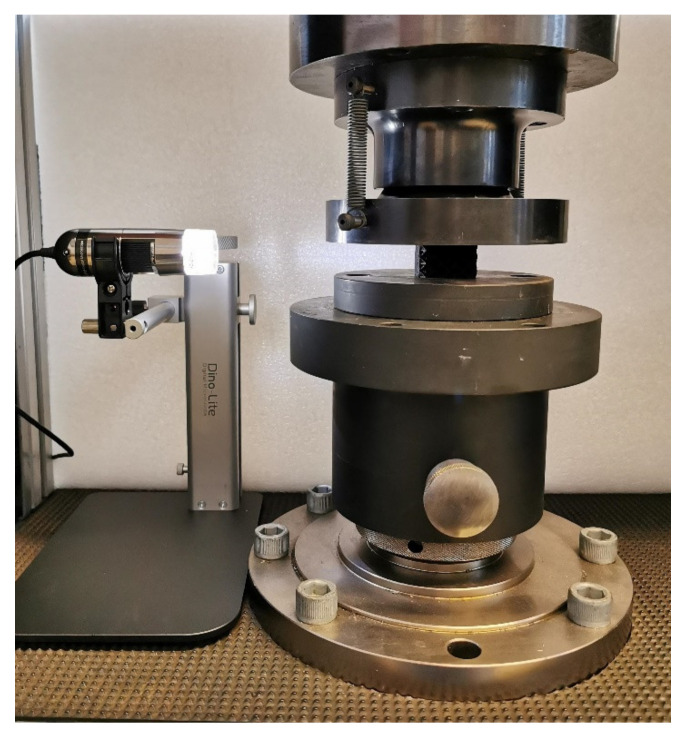
Compression test setup.

The cubic lattice structure specimens were subjected to compression tests in order to investigate the absorbing capabilities of the investigated cell geometries. A Zwick-Roell Z100 was used for the experimental tests by imposing a crosshead displacement of 1 mm/min. The tests were also recorded by using a Dynolite^®^ microscope, in order to compare the experimental failure mode with that found with numerical simulation. The tests were stopped manually when the densification was approached or for a compression displacement larger than 60% of the total specimen height. Figure 3 shows an image of the testing setup.

### 2.4. Numerical Model of the Lattice Structure

The compression tests of the cubic lattice structure specimens were simulated in the LS-DYNA environment with explicit time integration in order to investigate the collapsing mode and the efficiency of the structures. Furthermore, the comparison and validation of the numerical model with respect to the experimental results have allowed for the design of a crash absorber for automotive applications made of lattice structures.

Although the use of 3D elements would lead to a more accurate description of the lattice structure, a full-scale automotive component such as the crash absorber would require a significant computational effort. Therefore, in order to reduce the computational time, 1D Hughes–Liu beam elements of 1 mm length were used to discretize the lattice structure. The Hughes–Liu formulation with cross section integration is based on the transformation of the isoparametric 8-nodes element [27]. In order to guarantee the self-contact of the beams during the crushing phenomenon, the contact type *CONTACT_AUTOMATIC_GENERAL was retained.

The mechanical behavior of the lattice structures was simulated with an elastoplastic material law, namely *MAT_PIECEWISE_LINEAR_PLASTICITY. In accordance with [26], the Young modulus and the yield limit were 3510 MPa and 20 MPa, respectively, and the plastic field was described with the stress deformation curve reported in [26]. It must be noted that the mechanical properties of the printed parts depend on the microstructure of the filament. However, a correlation between the microstructure of the filament and the mechanical properties of the printed part can hardly be assessed, especially for complex structures such as lattice structures with beams characterized by small diameters. Indeed, together with the microstructure of the filament, the mechanical properties of the printed lattice structure parts were affected by multiple factors such as the process parameters, and, mainly, the manufacturing defects (e.g., variation of the diameter with respect to the nominal one, sharp reduction in the cross-section inducing a high stress-concentration) that form during the repeated fusions of the filament. For example, according to [28], the tensile strength of the filament is significantly different from that of the printed part. This is why an experimental calibration of finite element models is required for lattice structure components, since it would permit taking into account all the factors affecting the mechanical properties and the influence of defects, which, on the other hand, cannot be quantified by considering the properties of the filament.

Finally, two rigid walls, one fixed and one moving in accordance with a prescribed motion law, were retained to model the quasi-static compression test. In particular, in order to limit the inertia effect, the velocity of the moving rigid wall was smoothly increased up to a constant value.

## 3. Results of the Experimental Campaign and Numerical Investigations

In this section, the experimental results are analyzed with the aim of assessing how the energy absorption capability of the investigated lattice structures varied with the unit cell geometry parameters. Furthermore, the aim of this analysis was to provide useful indications for the design of components made of or filled with lattice structures. The experimental data were analyzed in a statistical framework. In Section 3.1, details on the methods and on the parameters considered for the analysis of the experimental data are provided. In Section 3.2 and Section 3.3, the results of the experimental tests carried out following *FP1* and the *FP2* are analyzed, respectively. Section 3.4 finally compares the FE model of the compression test to the experimental data.

### 3.1. Methods for the Analysis of the Experimental Data

In this section, details on the methods considered for the analysis of the experimental data are provided. This will help the reader to more easily understand the analysis carried out in Section 3.2 and Section 3.3.

**Figure 4:**
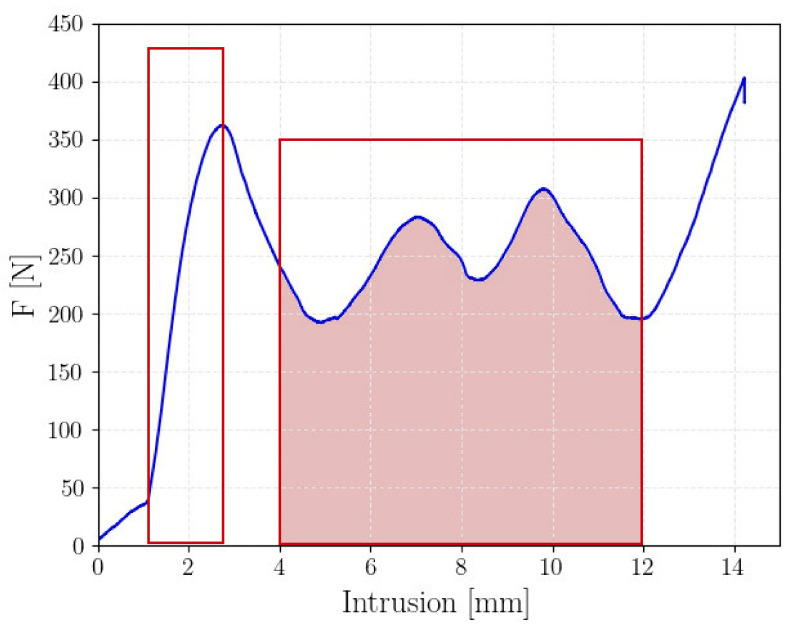
Example of a force–displacement curve acquired during the compression tests.

As specified in Section 2, compression tests were carried out to compare the absorbing capabilities of lattice structures with different geometries. Figure 4 shows a representative force–displacement curve of lattice structure specimens subjected to compression load (test A1B3).

According to Figure 4, the force–displacement curve shows an “elastic” region, with a rapid increment of the force up to a peak force, which was clearly recognizable. Thereafter, the force dropped down in the plastic region, and peaks and valleys alternated with a constant average value, each one corresponding to the cell failure. Finally, the densification started, with the force rapidly increasing. This is the common force–displacement curve found experimentally. It must be noted that in the first region (i.e., the initial part of the curve characterized by a slope smaller than that of the curve in the elastic region), the upper plate had already come into contact with the upper end of the specimen, but the compression on this surface was not uniform and incomplete.

In the following analysis, the following quantities were considered in the analysis for a proper comparison of the absorbing capabilities:

- Peak crushing force, PCF [N]: corresponds to the peak force, as indicated in Figure 4.
- Absorbed energy, AE [J]: corresponds to the whole area below the force–displacement curve up to the densification, computed through numerical integration (i.e., ∫Fdx, where F is the measured force and x the displacement of the cross bar of the testing machine).
- Specific energy absorption, SEA [J/g]: defined as the ratio between the energy absorbed up to the densification, AE, divided by the specimen mass, m (i.e., $SEA=\frac{AE}{m}$). The mass of each specimen was measured by using a digital balance with 0.01 g resolution. This normalized parameter is of utmost importance in this analysis, since a comparison based only on the AE would not permit considering the influence of the cell mass, which is, on the other hand, a fundamental parameter in lightweight applications. Accordingly, the SEA provides information on the absorbing capability efficiency and permits comparisons between cells with different characteristics and geometries.
- Mean Cushing Force, MCF [N]: represents the mean force in the plastic region, as indicated in Figure 4.
- Crush Force Efficiency, CFE [%]: the ratio between the MCF and the PCF and is expressed as a percentage value (i.e., $CFE=\frac{MCF}{PCF}$). It provides information on the crash efficiency. For CFE close to 100%, the difference between the PCF and MCF was limited, with the impact response close to the ideal response that allows for achieving the optimal energy absorption.

### 3.2. Experimental Results (FP1): Influence of the Beam Diameter and of the Cell Size

**Figure 5:**
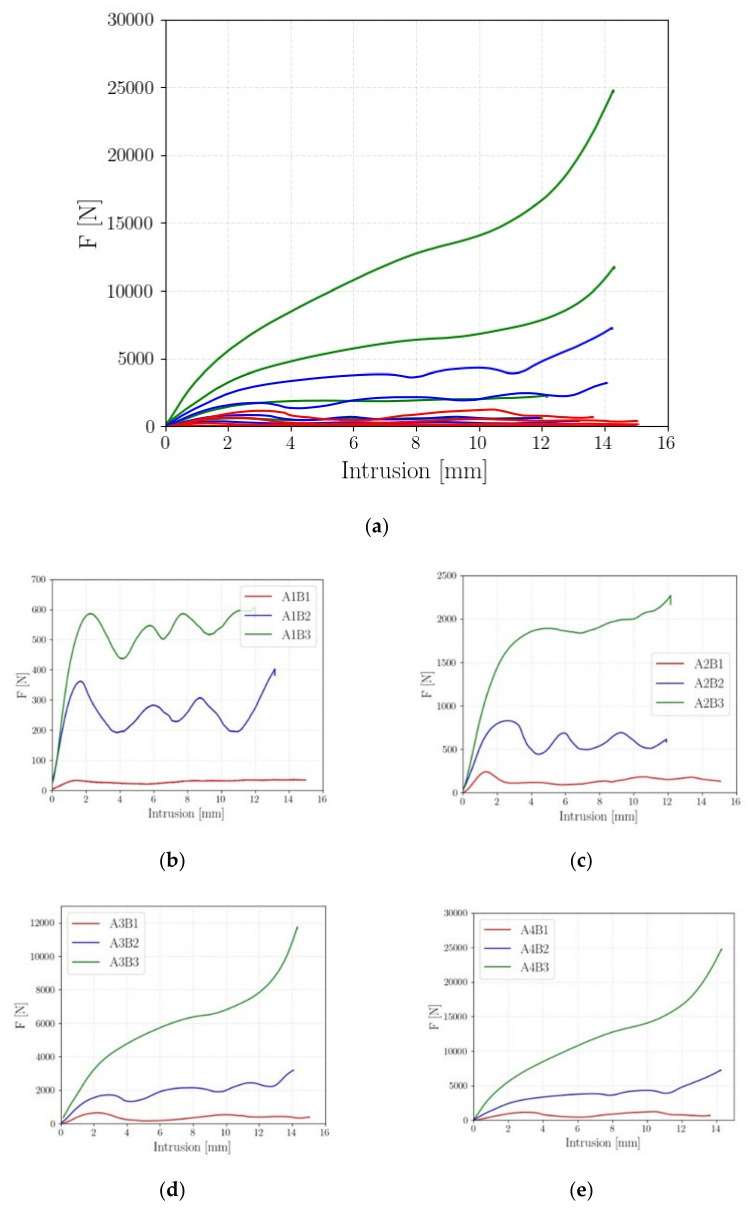
Experimental force–displacement curves for *FP1*: (**a**) one representative curve for each specimen condition; (**b**) force–displacement curves for d = 0.9 mm; (**c**) force–displacement curves for d = 1.2 mm; (**d**) force–displacement curves for d = 1.5 mm; (**e**) force–displacement curves for d = 1.8 mm.

In this section, the results of the *FP1* are analyzed in a statistical framework. The objective of this analysis was to investigate the influence of the beam diameter and the cell size, *l_cell_*, on the absorbing capabilities of the investigated cell geometry, selected in [20]. For each investigated combination of factors and levels (each line of Table 2), two tests were carried out. Figure 5 shows one of the force–displacement curves experimentally assessed for each of the investigated testing conditions. Figure 5a shows all the curves together. Figure 5b shows the force–displacement curves for d = 0.9 mm, Figure 5c for d = 1.2 mm, Figure 5d for d = 1.5 mm, and Figure 5e for d = 1.8 mm.

Figure 5 shows that the force–displacement curves for small *d* and large *l_cell_* had the typical trend of Figure 4, with peaks and valleys in the plastic region corresponding to the cell failures. On the other hand, for high cell densities (i.e., the small *l_cell_* and large *d*), the lattice structure specimens showed a foam-like behavior, with the peaks and the valleys associated with the cell failures tending to disappear and the force gradually increasing with the displacement in the material plastic region, up to the densification.

Table 3 summarizes the results of *FP1*: for each cell combination, the parameters described in Section 3.1 are reported. When the curves showed a foam-like trend, marked with the * symbol in Table 3, the MCF and of the CFE parameters could not be properly computed and are not reported. The average values are reported in Table 3, since the experimental data showed good repeatability. The percentage difference from the average value for the investigated parameters in Table 3 (computed as the difference between the average values and the experimental values, normalized with respect to the average values) was found to be generally smaller than 10%, being close to 20%, only for the A2B2 and the A4B1 tests.

In the A2B2 and A4B1 tests, the two experimental curves showed the same trend in the force–displacement curve, but one curve was below the other. Since the trend was the same, a different failure mode can be excluded to justify the larger difference between the two curves. Therefore, the large difference can be ascribed to the random distribution of defects in the lattice beam, which may have locally reduced the beam diameter, thus reducing its cross-section, or locally increased the stress concentration factor, enhancing the failure of a layer and, consequently, lowering the force–displacement curve. Indeed, even if the same process parameters have been set, a local reduction of the beam diameter may randomly occur and cannot be controlled. If a defect or a beam with smaller diameter randomly occurs in a critical region, a premature failure of one layer of the cell layer may occur, thus lowering the force–displacement curve.

As shown in Table 3, the absorbing capability efficiency increased with the cell density. Indeed, for the same level of factor B (cell size), the absorbed energy and, accordingly, the SEA, increased with the cell diameter. Similarly, for the same beam diameter, the absorbed energy and the SEA tended to increase with the cell density (i.e., the decrement of *l_cell_* provided higher values of the AE and the SEA). The PCF followed the same trend of the SEA, since it increased with the *d* and by reducing *l_cell_*. The CFE increased with the cell density. On the other hand, a clear trend was not found for the CFE by considering the cell diameter. Indeed, for the 2 *×* 2 configuration, the CFE reached its highest value for the smallest investigated diameter. For the 3 *×* 3 configuration, the CFE decreased when the diameter was increased from 0.9 mm to 1.2 mm, whereas it reached its maximum when the diameter was equal to 1.8 mm. In conclusion, the cell showing the best performance was the A4B3 cell, which had the highest SEA, the largest EA, and the highest PCF.

ANOVA analysis was performed to investigate, in a statistical framework, the influence of the *d*, the *l_cell_* factors, and their interactions on the SEA. The residuals of the ANOVA analysis were verified to follow a normal distribution, in order to check the applicability of the ANOVA. Moreover, Levene tests were carried out to verify the homogeneity of the variance for each combination of the groups. Table 4 shows the results of the ANOVA analysis. SS in the table refers to the sum of squares, as provided by the Minitab^®^ Software.

**Figure 6:**
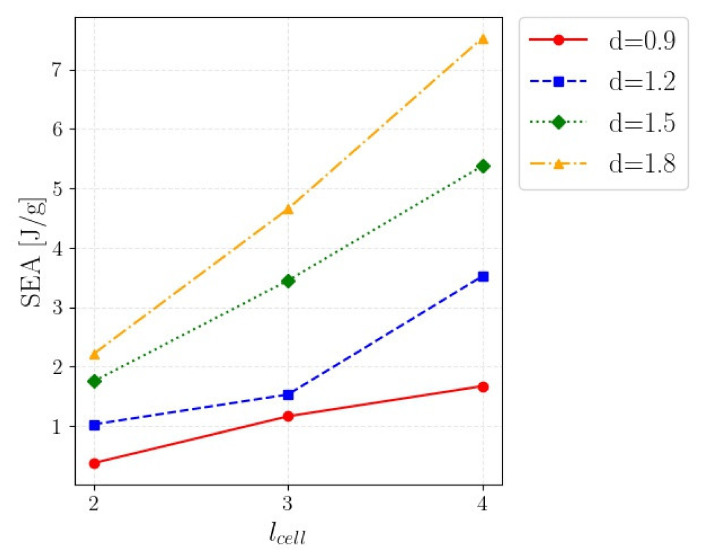
Interaction plot showing the average SEA with respect to the parameter *l_cell_* for the investigated *d*.

As expected and as shown in Table 4, the factors *d* and *l_cell_* were statistically significant, since the computed *p*-values were close to 0. The interactions were also statistically significant, with a *p*-value close to 0. This was confirmed by the interaction plot in Figure 6, showing the average SEA with respect to *l_cell_* for different beam diameters *d*. Indeed, according to Figure 6, the absorbing efficiency for the investigated cells increased with *n* and *d*. The increment of the SEA was larger for the largest *n* and the largest *d*, confirming the influence of the interactions.

To conclude, this analysis showed that the larger the cell density (i.e., large cell diameters with small cell sizes), the larger the efficiency in energy absorption. Accordingly, high-density cells should be employed to increase the energy absorption of the components. On the other hand, filling parts with high-density cells if the energy to be absorbed is not high (or filling the whole component if some regions are unloaded) would induce an undesired increment in the mass, which is to be avoided in a lightweight design. In these cases, an infill pattern with lattice structures with variable density (cell size and beam diameter) can be the optimal strategy to obtain high energy absorption with limited weight.

### 3.3. Experimental Results (FP2): Influence of the Beam Diameter and of the Specimen Size

The objective of this second analysis was to verify whether the specimen size affects the absorbing capability. This analysis is fundamental, since components are generally manufactured with many layers of a unit cell, but the design is generally based on simulations or on experimental tests carried out on a single cell or on a limited number of cells [20]. For example, in [29], the failure mode of aluminum specimens depended on the number of cells. This could have significant implications on the design of the final component, since the failure mode can affect the absorbing capability of the part.

**Figure 7:**
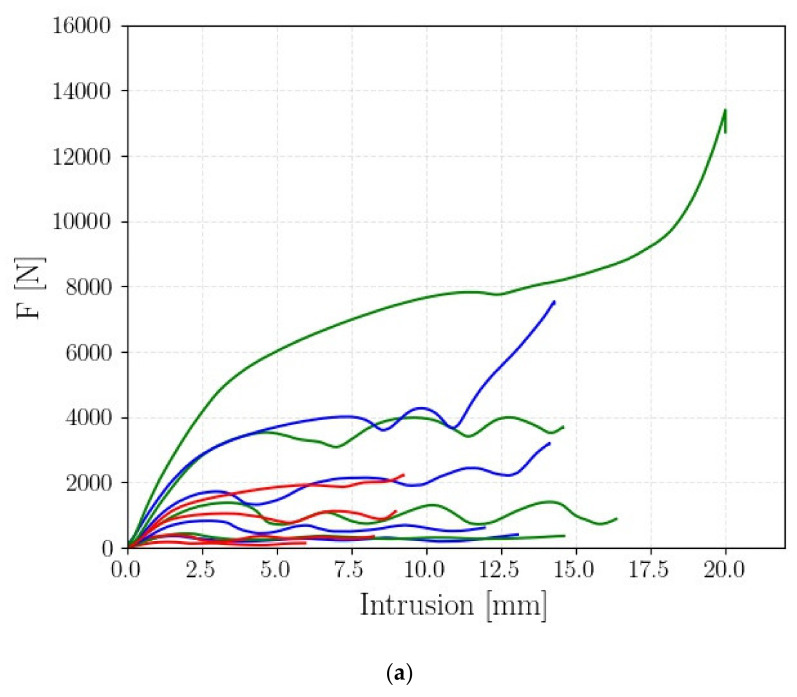

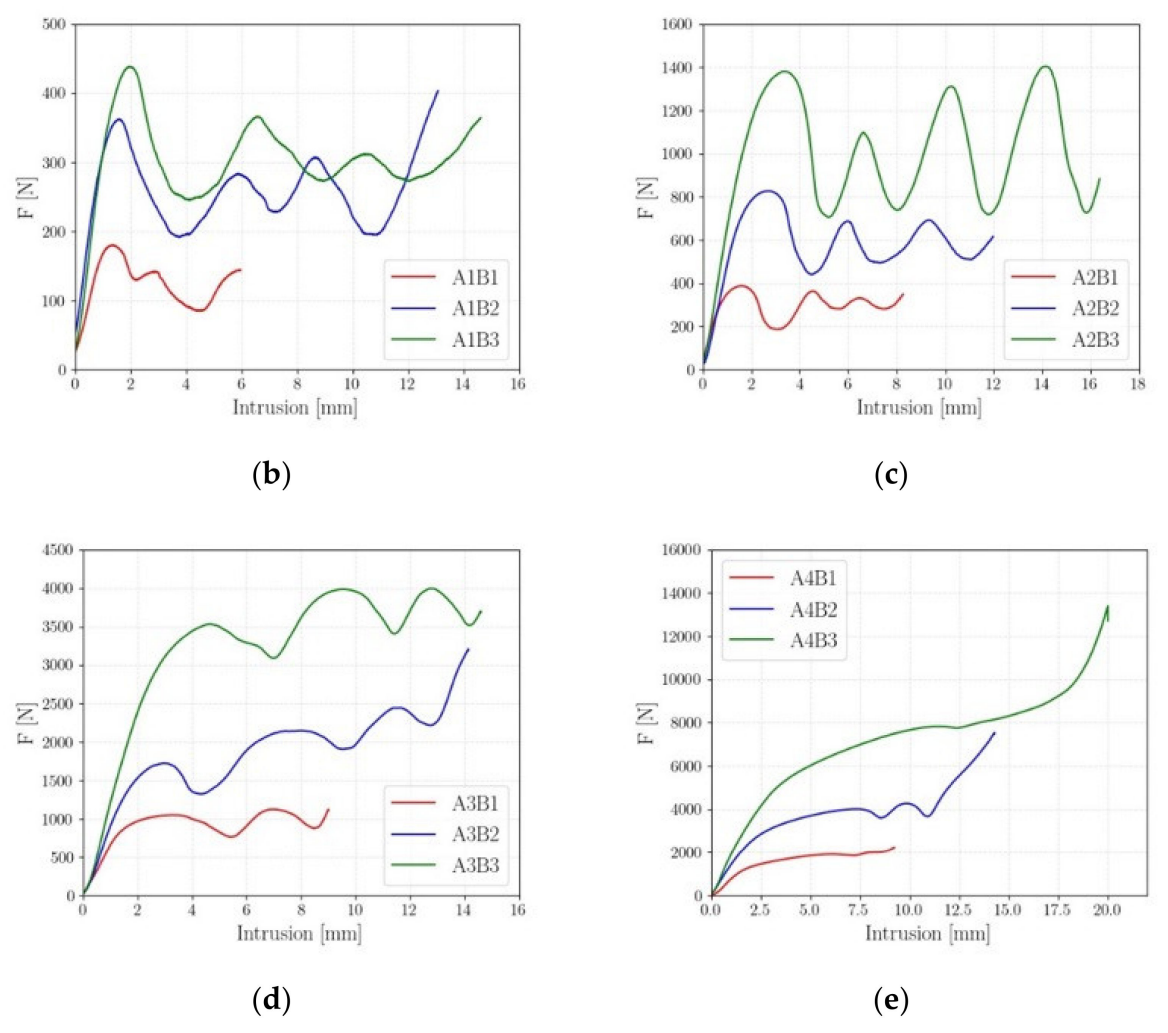
Experimental force–displacement curves for *FP2*: (**a**) one representative curve for each specimen condition; (**b**) force–displacement curves for d = 0.9 mm; (**c**) force–displacement curves for d = 1.2 mm; (**d**) force–displacement curves for d = 1.5 mm; (**e**) force–displacement curves for d = 1.8 mm.

As illustrated in Section 2, the FP2 scheme corresponded to the FP1 scheme. However, in this case, rather than the final specimen volume, the geometry of the unit cell was bee kept constant and equal to that considered in [20]. Accordingly, the specimen size varied when n was varied. Figure 7 shows one of the force–displacement curves experimentally assessed for each investigated specimen. Figure 7a shows all the curves together. Figure 7b shows the force–displacement curves for d = 0.9 mm, Figure 7c for d = 1.2 mm, Figure 7d for d = 1.5 mm, and Figure 5e for d = 1.8 mm.

As shown in Figure 7, the force–displacement curves showed the “peak–valley” trend in the plastic region for d up to 1.5 mm. For d = 1.8 mm, the foam-like trend was, on the other hand, found. This behavior is different from that found for FP1 and in Figure 5, for which the foam-like trend was also found for d = 1.2 mm and d = 1.5 mm. This difference can be justified with the different cell sizes, which was fixed in FP2, whereas it varied in FP1.

Table 5 summarizes the results of the *FP2*; for each tested specimen, the parameters described in Section 3.1 are reported. For *FP1*, two tests for each investigated condition were carried out and the mean value was reported. In addition, for *FP2*, the experimental data showed good repeatability, with the percentage difference from the average value for the investigated parameters in Table 5 generally smaller than 10% and close to 20% only for the A2B2 and A2B3 tests.

According to Table 5, the PCF and the EA parameters tended to increase with the number of cells and the cell diameter, as expected. A different behavior, on the other hand, was found for the CFE and the SEA. Indeed, the CFE showed slight variations with *n*, whereas it increased with the diameter *d*. The SEA increased with *d*, but the experimental data did not show a clear trend for the number of cells. For example, for d = 0.9 mm, the SEA for *n = 2* and *n = 3* was close and it decreased for *n = 4*. In order to confirm these results, an ANOVA analysis was also carried out. For the *FP1* testing plan, the applicability of the ANOVA was verified by analyzing the normality of the residual of the ANOVA and through Levene tests. Table 6 shows the results of the ANOVA analysis.

**Figure 8:**
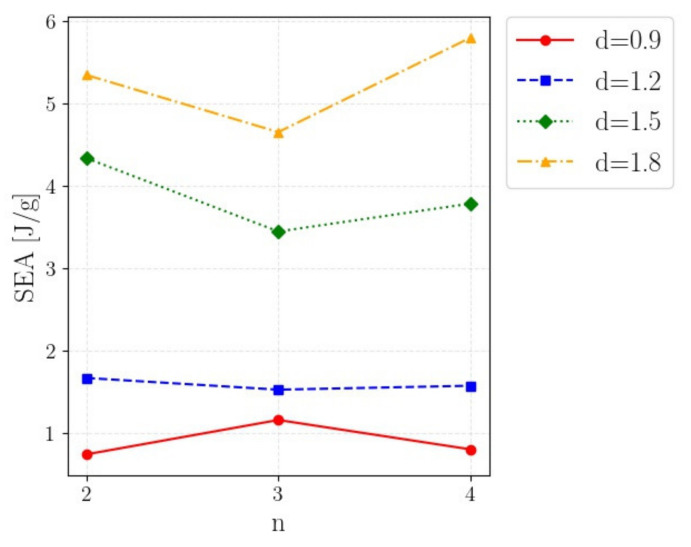
Interaction plot showing the average SEA with respect to the parameter *n* for the investigated *d*.

The ANOVA analysis confirmed that the beam diameter strongly influenced the SEA, whereas the number of cells was not a statistically significant factor, since the associated *p*-value was above 0.2. Similarly, the interactions were not statistically significant for a significance level up to 10%. The interactions plot in Figure 8, showing the average SEA with respect to the parameter n for the investigated d, helps to visualize these results.

As shown in Figure 8, the average SEA did not increase with the number of cells. For diameters up to 1.5 mm, the trend was rather flat. On the other hand, for d = 1.8 mm, the SEA increased with n, probably due to the transition from the “cell failure” modes to the “foam-like” failure mode. These results confirm that, for the investigated material and for a fixed unit cell geometry, the number of cells did not significantly influence the SEA, even if slight interactions could be seen in Figure 8 (justifying the *p*-value associated with the interactions close to 10%). This means that the absorbing capabilities of the investigated cell can be properly assessed through tests on specimens with a limited number of cells (thus permitting to reduce the manufacturing time) and that the results obtained in FP1 are valid and can be used for the design of components with lattice structures.

### 3.4. Numerical Investigations: Comparison with the Experimental Results

As highlighted in the previous sections, according to the diameter size and unit cell size, the lattice structure showed either a gradual crushing of the cell layers, whose resulting force–displacement curve is characterized by peaks and valleys or a foam-like crushing behavior where peaks and valleys tend to disappear. In order to validate the finite element model, typical cases of gradual collapse and foam-like collapse were considered. The structures are the A2B2, which is common to the two factorial plans and showed a very gradual collapse, and the A4B3 of the FP1, which instead showed a foam-like force–displacement curve.

**Figure 9:**
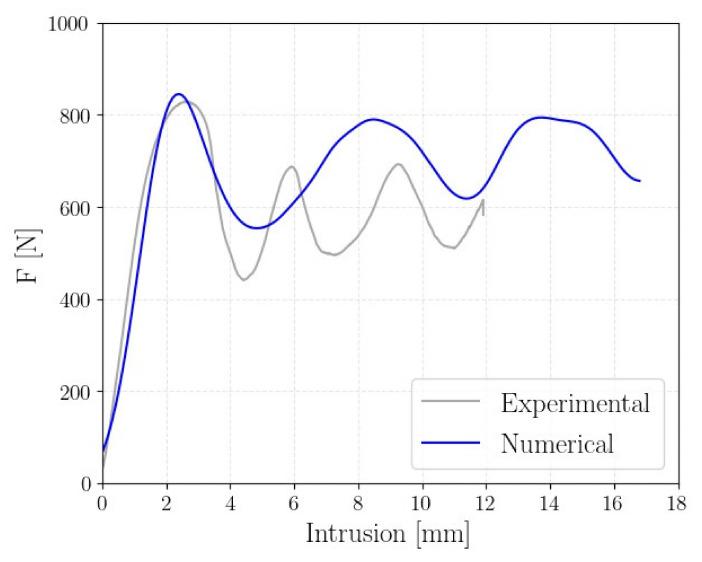
Comparison of the experimental and numerical force–displacement curves of the A2B2 lattice structure.

Figure 9 reports a comparison of the experimental and numerical force–displacement curves of the A2B2 structure.

**Figure 10:**
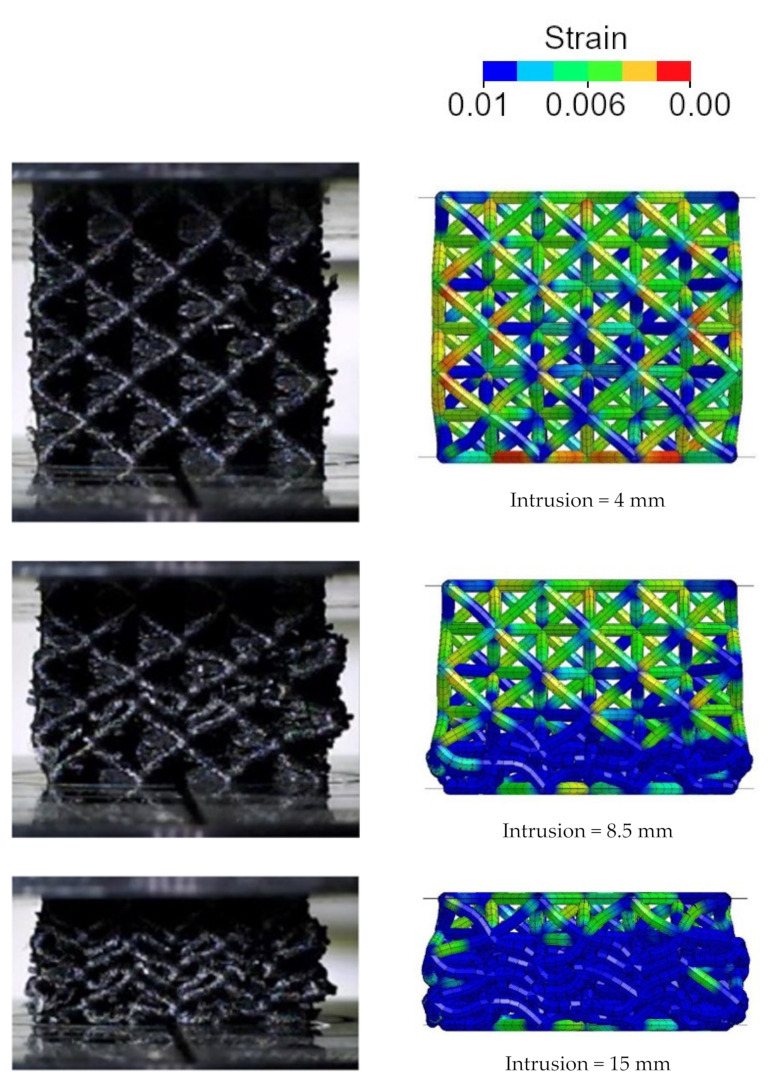
Experimental and numerical compression sequence of the A2B2 lattice structure: plastic strain contour plot in the numerical model.

As shown, the numerical model well replicates the force–displacement curve characterized by significant peaks and valleys. The discrepancy in the length of the peaks and valleys can be mainly attributed to geometrical imperfections of the specimen. The nonperfect geometry of the additive manufactured beams facilitates the local buckling of the cell. The peaks and valleys of the crushing force are indeed the results of the gradual collapse of the lattice structure. Figure 10 shows the numerical collapsing mode in comparison with the experimental test. In particular, the strain contour plot is shown.

The finite element model shows that, as the compression proceeds, each layer of cells progressively yields, thus leading to the peaks and valleys. Furthermore, it is worth noticing that, at the end of the test, the whole lattice specimen yielded, thus confirming the efficiency of the structure.

**Figure 11:**
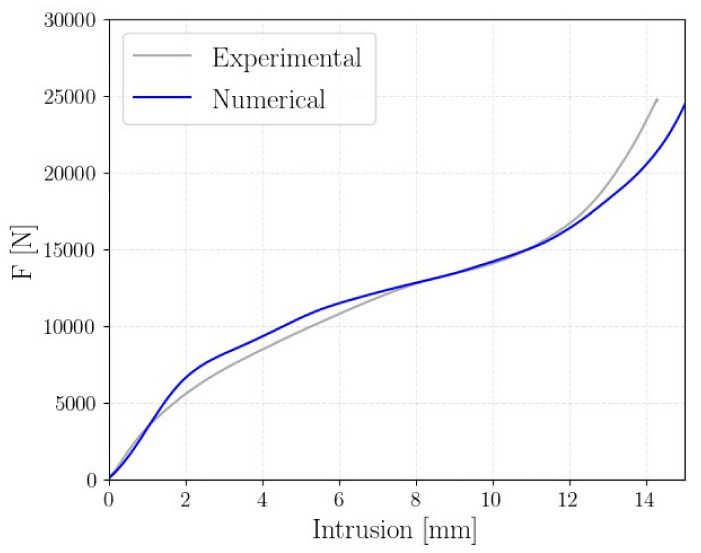
Comparison of the experimental and numerical force–displacement curves of the A4B3 lattice structure.

In Figure 11, the numerical results of the A4B3 structure (i.e., the best performing structure according to the results of Section 3.2 and Section 3.3) are compared to the experimental data.

**Figure 12:**
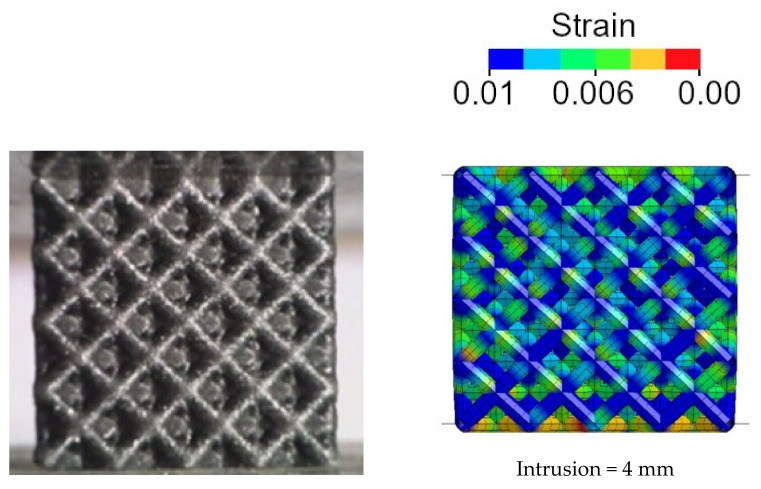

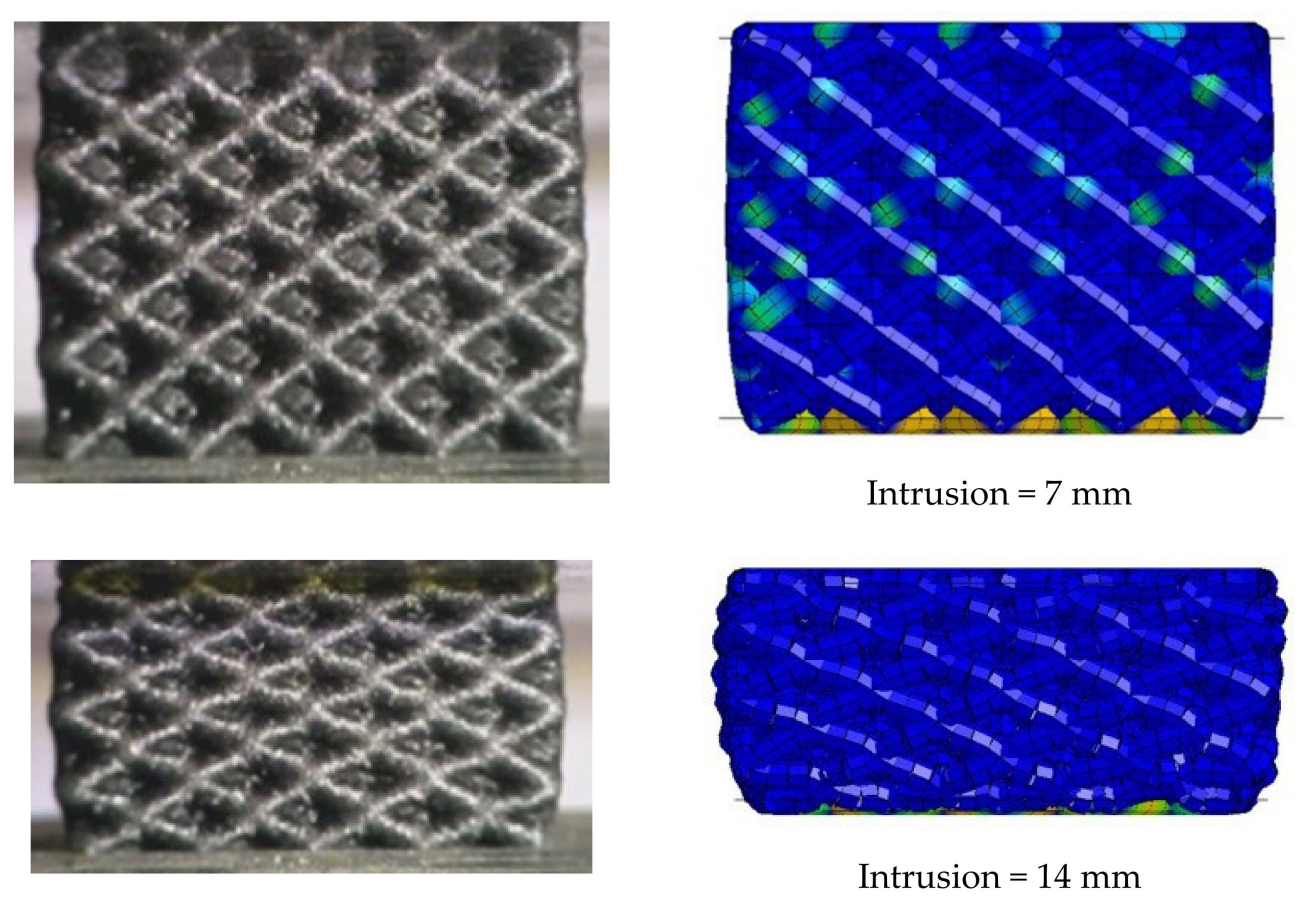
Experimental and numerical compression sequence of the A4B3 lattice structure: plastic strain contour plot in the numerical model.

In this case, the structure failed with a foam-like force–displacement curve. Additionally, in this case, the finite element model was able to well replicate the mechanical behavior of the lattice structure. The very good agreement between the numerical and experimental results can also be appreciated in Figure 12 where the experimental and numerical collapsing modes are compared. Regarding structure A2B2, the strain contour plot is shown.

As shown, the sequence of collapse founded experimentally was well replicated by the simulation. Unlike the A2B2 structure, the layers were continuously compressed until densification occurred, without showing the gradual failure of each cell layers. As shown through the strain flow, the whole structure simultaneously yielded. Therefore, thanks to the peculiar geometrical configuration and to the material characteristics, the whole material of the lattice structure is strongly involved in the compression, thus confirming the high efficiency of this structure.

In conclusion, the numerical analysis showed that, regardless of the collapsing mode, the whole material of the lattice specimens yielded. The high effectiveness of the unit cell defined in [20] was thus confirmed. Furthermore, in accordance with the experimental results, the numerical model showed that a foam-like collapse mode was more efficient as the whole structure simultaneously yielded. Regarding the numerical model, it is worth noticing that even though the intrinsic variability of the manufacturing process affected both the resulting geometry and the material properties, satisfactory accuracy can be obtained even through 1D elements. More sophisticated analyses of the microstructure or of the material properties, which might account for local geometry variations or for the dependency of the material on the printing direction, can be useful to investigate local material failures and their correlation with the filament and/or printed microstructure. However, the simplified model here proposed and based on 1D elements was able to correctly capture the global mechanical behavior of the structure while consistently saving the computational time. Simulations of the lattice structures approximately took from 100 s to 600 s, depending on the dimensions of the structure, on a desktop computer with Intel Core i7-8700 (3.2 GHz) and 32 GB of RAM.

## 4. Design of a Crash Absorber

The preliminary design of a crash absorber for automotive applications was addressed in accordance with the experimental tests and once the numerical model was validated. The goal of this component was to absorb, through plastic deformation, the kinetic energy in low-speed impact events (15 km/h), preventing injuries to passengers or consistent damage to other components such as the radiator.

To assume a realistic design scenario, the crash tube employed in the Toyota Yaris was considered, and the geometrical constraints in terms of envelope (235 mm *×* 89 mm *×* 109 mm) were also taken into account. Considering a C-segment vehicle with a mass of 1360 kg impacting at 15 km/h with a 40% of overlap in accordance with the RCAR low speed structural crash protocol [30], the crash tube has to absorb the kinetic energy of 11.8 kJ. The reference structure was a thin-walled tube made of steel with a constant thickness of 1.8 mm. The cross section varied along the tube as in tapered structures. The finite element model is available at their website [31]. In particular, Belythsko–Tsai four node elements were used to model the thin-walled tube. In our simulation, the bottom part of the structure was constrained and a rigid wall with an initial kinetic energy equal to 11.8 kJ was considered.

Section 3 showed that the highest specific energy absorption was obtained for the densest structure (i.e., the one with the largest diameter and the smallest cell size). The lattice structure A4B3 of FP1 was therefore retained in the design of the crash absorber. However, as shown in Section 3.2, this lattice structure showed a foam-like behavior. Therefore, considering the results of FP2, which confirmed that the absorbing capabilities of the cell can be properly assessed with tests on specimens with a limited number of cells, a crash absorber obtained by replicating the lattice structure A4B3 of FP1 would show a foam-like force–displacement curve. The ideal crushing force is instead characterized by a constant trend in the whole crushing phenomenon. Furthermore, depending on the dimensions, a structure made by simply replicating the lattice structure A4B3 of FP1 might fail for a global buckling, thus consistently reducing its energy absorption capabilities.

**Figure 13:**
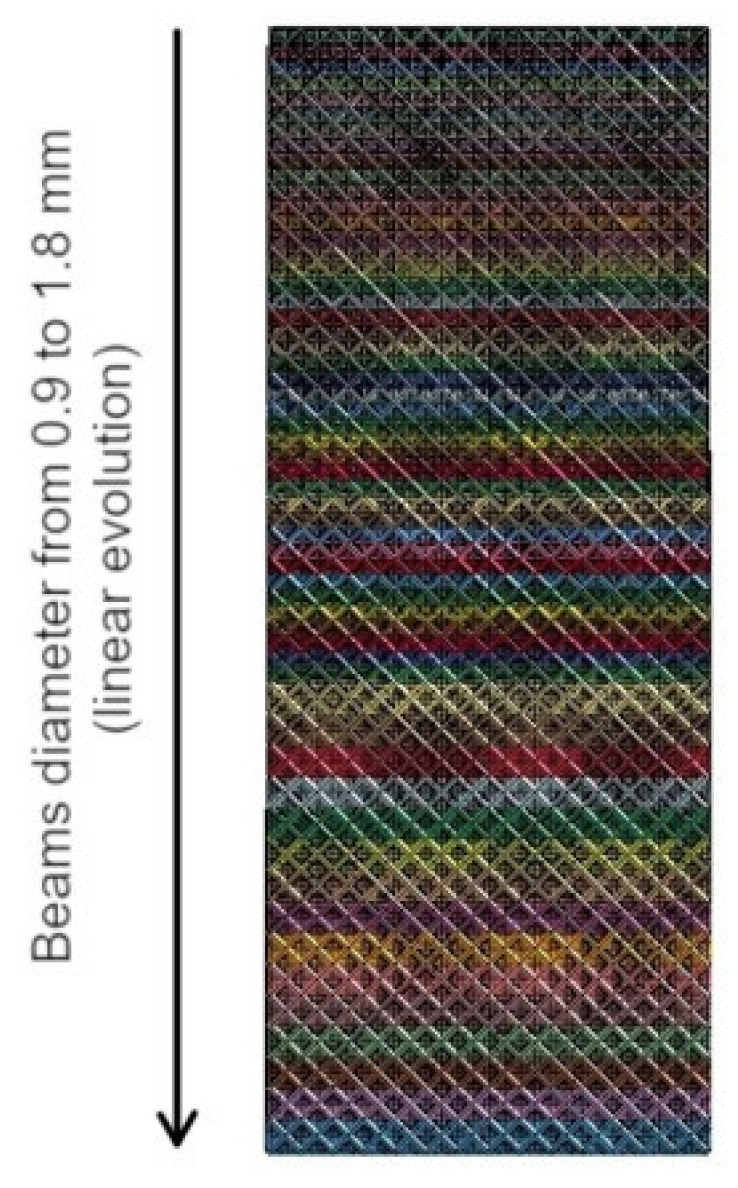
Geometry of the lattice crash absorber: linear variation of the beams’ diameter.

A crash tube with a variable diameter along the tube length was retained, in order to prevent the global buckling and to achieve a smooth force–displacement curve. In particular, a linear increase in the diameter of the cells was considered. Figure 13 shows the geometry of the retained crash tube.

**Figure 14:**
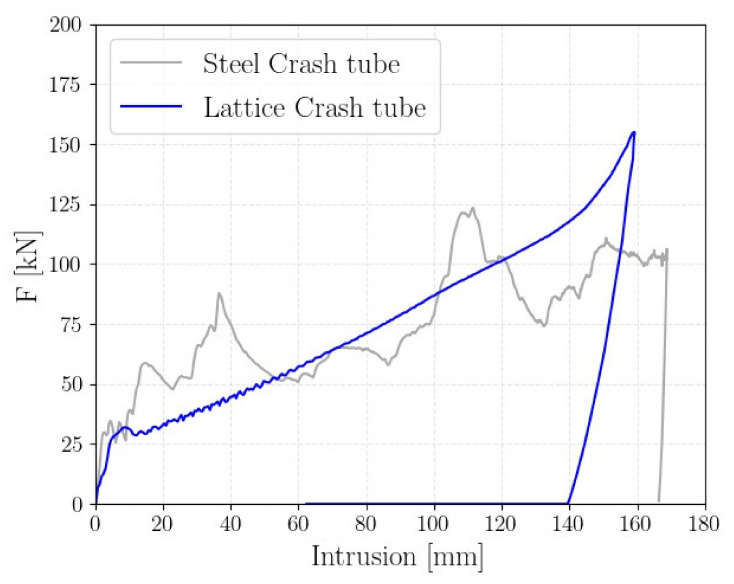
Comparison of the force–displacement curve between the reference crash tube and the lattice-based crash tube.

The crash absorber had dimensions of 230 mm *×* 90 mm *×* 90 mm, in accordance with the envelope constraints. The linear variation of the diameter extended for 180 mm. This allowed us to obtain a total mass of the component equal to 0.795 kg, which corresponded to a mass saving of almost 25% with respect to the reference structure. Figure 14 shows the comparison of the force–displacement curves of the reference and lattice crash tubes.

According to Figure 14, the crushing force was very smooth, without the typical peaks and valleys. The final peak of the force indicates that in the final phase of the compression, a slight densification occurs. The linear increase in the diameter acts as a trigger, thus preventing the global buckling and facilitating the gradual crushing of the tube. Additionally, as the diameter increases, the resistance to impact loads increases, thus leading to a tapered-like crush response, with the force linearly increasing as the intrusion proceeds. It is also worth noticing that the maximum intrusion of the lattice structure was 158 mm, while the maximum intrusion of the reference component was almost 170 mm. Therefore, a 7% reduction in the maximum intrusion was achieved with the proposed structure, which represents another important advantage for the passengers and components’ safety.

It is worth noticing that the SEA of the crash absorber was 14.8 J/g, which was higher than the value obtained for the cell A4B3 reported in Table 3 of 7.53 J/g. The consistent increase in the SEA can be attributed to the densification of the cells that, once crushed, still absorb energy by densification. In this regard, the use of detailed models, which account for both geometrical and material variations, appears even less convenient in the design of an energy absorber. Rather, an experimental calibration of the simplified finite element models is the most preferable approach for these applications.

The promising results of the lattice crash tube demands further optimization and investigation. First, the strain-rate dependency of the retained carbon nylon should be considered for a more accurate description of the crashworthiness of the component. Regarding the densification, it is worth highlighting that this mechanism appears very effective for energy absorption and requires further investigations to maximize the advantages of lattice structures. Finally, the printing time of the lattice structures can be high. Therefore, future work should account for the manufacturing process in the design of the crash tube, in order to achieve a fully feasible solution.

## 5. Conclusions

In this paper, the influence of the beam diameter and cell size on the energy absorption capability of a lattice cell, whose morphology was identified in a previous work, was investigated. The lattice cells were made of carbon nylon by the fused deposition modeling (FDM) process and subjected to quasi-static compression tests. In particular, two multilevel factorial plans for investigating the compression response were performed: in the first, the global volume of the specimen was constant and the dimension of the unit cell was varied according to the number of cells; the second factorial plan assumed constant dimensions of the unit cell and the number of cells was increased with no volume constraints. In both factorial plans, the compression tests were repeated for different beam diameter. The mechanical behavior of the lattice structures was also investigated through a numerical model validated on the experimental results. The following conclusions can be drawn:

- according to both factorial plans, the larger the diameter, the higher the specific energy absorption (SEA) of the structure. In particular, according to the first factorial plan, as the size of the unit cell decreased (i.e., the density of the structure increases), the SEA increased;
- the second factorial plan also showed that for the investigated material and for a fixed unit cell geometry, the number of cells did not significantly influence the SEA. This means that the absorbing capabilities of the investigated cell can be properly assessed through tests on specimens with a limited number of cells (thus permitting a reduction in the manufacturing time);
- the numerical model showed that 1D elements can describe the mechanical behavior of the lattice structures with satisfactory accuracy. In energy absorption investigations, the use of 1D elements is particularly convenient as it allows one to consistently save on computational effort without affecting the accuracy of the results. Nevertheless, detailed analyses of the microstructure of the filament and of the printed part (e.g., through DSC/DMA), can be useful to investigate local material failures and their dependency on the manufacturing process. Furthermore, the numerical model highlighted that the collapse (i.e., local buckling of cell layers or foam-like failure) is governed by the unit cell geometry (i.e., diameter and length of the beams). However, independent of the typology of collapse, the whole lattice specimen yielded, confirming the efficiency of the structure; and
- based on the experimental and numerical results, the preliminary design of an automotive crash absorber made of a lattice structure was proposed. For a C-segment vehicle, the results showed that a mass saving of about 25% could be achieved through the proposed crash absorber with respect to the standard vehicle component made of steel. Additionally, the lattice crash tube had a smaller envelope and the maximum intrusion was 7% smaller.

## Figures and Tables

**Table 1:** Factors A and B and the levels considered for assessing the influence of the cell parameters on the absorbing capabilities of the investigated cell.

Levels	1	2	3	4
Factor A, beam diameter d	0.9 mm (0.8 mm)	1.2 mm (1.1 mm)	1.5 mm (1.4 mm)	1.8 mm (1.65 mm)
Factor B, number of cells, n	2 × 2	3 × 3	4 × 4	

**Table 2:** Multilevel factorial design considered to assess the influence of the beam diameter (Factor A, *d*) and the cell number (Factor B, *n*).

Test Identification	Levels	
	Factor A, d	Factor B, n
A1B1	1	1
A1B2	1	2
A1B3	1	3
A2B1	2	1
A2B2	2	2
A2B3	2	3
A3B1	3	1
A3B2	3	2
A3B3	3	3
A4B1	4	1
A4B2	4	2
A4B3	4	3

**Table 3:** Results of *FP1*: Comparison of the absorbing capabilities of the investigated cells.

Test Identification	AE [J]	SEA [J/g]	MCF [N]	PCF [N]	CFE [%]
A1B1	0.4	0.38	29	39	76%
A1B2	2.6	1.16	221	305	73%
A1B3	5.6	1.67	541	608	89%
A2B1	2.0	1.03	132	233	62%
A2B2	5.4	1.53	483	691	70%
A2B3	20.3	3.52	1883	2045	92%
A3B1	5.3	1.75	383	653	59%
A3B2 *	19.9	3.45	/	2441	/
A3B3 *	48.5	5.38	/	6541	/
A4B1	9.0	2.21	725	1138	63%
A4B2	36.3	4.65	3973	4293	93%
A4B3 *	90.1	7.53	/	14,050	/

**Table 4:** Results of the ANOVA for the *FP1* testing plan.

Factors	DoF	Adjust SS	Adjust MS	*p*-Value
*d*	3	0.000041	0.000014	0.000
*l_cell_*	2	0.000032	0.000016	0.000
*d l_cell_*	6	0.000008	0.000001	0.000
*Error*	9	0.000002	0.000000	
*Total*	20	0.000068		

**Table 5:** Results of the *FP2*: Comparison of the absorbing capabilities of the investigated specimens.

Test Identification	AE [J]	SEA [J/g]	MCF [N]	PCF [N]	CFE [%]
A1B1	0.6	0.75	198	180	68%
A1B2	2.6	1.16	222	305	73%
A1B3	3.9	0.81	317	458	69%
A2B1	1.9	1.67	247	338	73%
A2B2	5.4	1.53	485	691	70%
A2B3	13.1	1.58	958	1323	72%
A3B1	8.2	4.34	1026	1098	93%
A3B2	19.9	3.45	/	2954	/
A3B3	51.9	3.78	3680	3577	97%
A4B1	12.9	5.34	/	1877	/
A4B2	36.3	4.65	3975	3920	97%
A4B3	106.7	5.79	/	8196	/

**Table 6:** Results of the ANOVA for the FP2 testing plan.

Factors	DoF	Adjust SS	Adjust MS	*p*-Value
*d*	3	0.000066	0.000183	0.000
*n*	2	0.000000	0.000000	0.238
*d∙n*	6	0.000002	0.000001	0.129
*Error*	10	0.000001	0.000000	
*Total*	20	0.000072		

## Data Availability

The data presented in this study are available on request from the corresponding author.
